# Survey of the demand for care services for older people and the training needs of their care workers: a cross-sectional study in Northeast China

**DOI:** 10.1186/s12912-022-00809-1

**Published:** 2022-01-19

**Authors:** Shuqin Li, Jun Zhang, Yan Liu, Ai-ping Wang, Guixing Qiu

**Affiliations:** 1 Department of Nursing, First Affiliated Hospital of China Medical University, Liaoning Shenyang, China; 2 Department of Orthopedics, Peking Union Medical College Hospital, Beijing, China

**Keywords:** Geriatric care, Old-age service, Service demand, Care workers, Training needs

## Abstract

**Background:**

The older population is increasingly utilizing professional healthcare services, while the requirements for caregivers are becoming more demanding. Therefore, it is important to be mindful not only of the service needs of older people but also to consider the training needs of their care workers. The present study aimed to investigate the care service needs for older people and the training needs of their care workers.

**Methods:**

A cross-sectional questionnaire was used to survey 589 residents of 6 nursing homes and 2 geriatric hospitals, 415 medical staff from 7 geriatric hospitals, 5 nursing homes, and 1 community institution, and 372 nursing assistants from 21 nursing institutions in northeast China.

**Results:**

The service with the greatest demand and that with which users were most satisfied was regular visits by healthcare personnel, which was the case for 87.27% of the care recipients. Of the medical staff, 75.42% had training needs related to geriatric healthcare, while the most requested training content was the comprehensive assessment of old people. The most requested method for the delivery of training was by self-study online video courses. Of nursing assistants, only 53.4% had obtained the relevant practicing certificate. While 83.6% participated in relevant training, 86% expressed the need for additional training. The majority of this category of staff wished to receive training in everyday care routines, and the majority wanted to learn by way of practical training.

**Conclusions:**

The care needs of the older population are diverse, and the work performed by healthcare personnel is increasing in scope. The existing training system for such care personnel is not perfect, and the demand for training is high. Existing training methods and content require improvement.

## Introduction

It is clear that the global population is aging. Over the period 2015-2050, it is estimated that the proportion of the global population aged 60 and above will almost double, from 12–22% [1]. In 2020, 180 million of the population were aged 65 and above in China, accounting for approximately 13% of the total population. It is estimated that by 2035, the number aged 65 and above in China will reach 310 million and 380 million by 2050, accounting for 22.3% and 27.9% of the total population, respectively [2]. With such numbers increasing inexorably, the numbers of older people requiring long-term care will also increase significantly. In addition, the overall numbers of disabled people are increasing rapidly in China, and are estimated to grow from 43.75 million in 2020 to 91.4 million in 2050 [3], while approximately 75% of the older population currently suffers from at least one chronic disease [4]. Satisfying the growing needs of this category of individuals in terms of health and care services represents a considerable challenge [5, 6].

At present, care services for older people include informal care provided by family members and formal care provided by institutions such as nursing homes. China’s long-standing one-child policy combined with the aging of the population has led to a rise in the dependency ratio, a heavy burden on the young, and a decline in the ability of families to provide adequate care for their aging family members [7, 8]. In addition, China’s expansion of urbanization and large-scale migration of the population make balancing work and providing care for older relatives a challenge for the young. This has resulted in increased numbers of empty nesters in urban and rural areas. Therefore, the older population increasingly has formal care requirements [9]. In addition, with improvements in material and cultural living standards, public opinion and the need for geriatric care have also changed. Traditional informal care services cannot provide a high quality of life for older people, and so they are increasingly utilizing professional geriatric care services [10–12].

As aging of the population becomes more acute, the number of the older population that is disabled, has dementia, or is solitary has increased. The care model of combined medical care and nursing has become an important method of servicing a population that is aging, requiring a higher level of specialization in geriatric care [13, 14]. High-quality care for older people must be provided by a professional team, but the formation of such care teams in China is far from perfect, especially regarding nursing assistants, reflected by their low level of education and lack of professional knowledge [15]. Therefore, improving the overall quality and skill level of care teams is an important step to providing adequate support. Due to the rapid expansion of the aging population in China, research on the training of healthcare personnel continues to be explored. There is a strategic significance in understanding the current situation and the training demands of healthcare personnel for older people. The key issues need to be identified and the basis for improvements in training systems for healthcare personnel for older people in China require analysis for the future.

According to international standards, aging societies are defined as those in which the population aged 60 or above in a country or region accounts for more than 10% of the total population or more than 7% of the population aged 65 or above [16]. The latest national census data indicate that the proportion of the population aged 65 or above in Northeast China is 16.39%, higher than the national average [17]. The present study will analyze the population in Northeast China to investigate their needs for geriatric care services and the training needs of the healthcare personnel, aiming to represent a reference for improvement in the quality of the care services and to satisfy the diverse needs of older people.

## Materials and methods

### Study design and participants

A cross-sectional study was conducted in July and August 2020 among geriatric hospitals, geriatric nursing homes, and communities in northeast China. The directors of each geriatric institution were contacted and asked to include as many participants as possible using a convenient sampling method. A total of 589 residents from 6 geriatric nursing homes and 2 geriatric hospitals, 415 medical staff from 7 geriatric hospitals, 5 geriatric nursing homes, and 1 community institution, and 372 nursing staff from 21 geriatric nursing homes were included in the study. Recipients of care were included if they met the following criteria: ① Aged 65 and above; ② Possessing clarity of thinking, ability to express themselves and without any apparent difficulty in communication; ③ Volunteered to participate in the study and provided signed informed consent. Exclusion criteria for older people were as follows: the presence of acute disease of the heart, brain, or kidney, or other disease. The inclusion criteria for medical staff were: ① Possession of a relevant professional qualification certificate; ② Working in that institution for more than 6 months; ③ Normally undertaking professional geriatric medical care (treatment / nursing / rehabilitation / health management, etc.). Exclusion criteria for medical staff: ① Those that had not been on duty for more than 1 month; ② Interns or trainees; ③ Personnel not directly involved in front-line work. Inclusion criteria for nursing staff: ① Those working in that institution for more than 3 months; ② Normally caring for old people. Exclusion criteria for nursing staff were as follows: (1) Those that had not been on duty for more than 1 month; ② Interns or trainees. The study was approved by the ethics review committee of Peking Union Medical College Hospital, Chinese Academy of Medical Sciences.

### Instruments

Self-designed questionnaires were used in the present study.

#### Questionnaire for care recipients

The questionnaire included basic personal information, their requirements for healthcare services, and the status of the services they receive. Personal basic information included age, gender, level of education, types of chronic disease, level of self-care (Not dependent: no requirement for others to provide care services; Mildly dependent: others required to take care of a small proportion of daily living needs; Moderately dependent: most daily living needs require others to provide care; Severely dependent: all needs of daily living require the care of others), evaluation of their current physical condition (on a scale from 0 to 100, where 0 is very poor and 100 is very good), their current type of care institution, reasons for choosing this category, and other care services they wished to receive, etc. Their requirements and the status of the healthcare services they received included five aspects of healthcare services (3 items), medical care services (3 items), rehabilitation guidance services (2 items), psychological/spiritual support services (1 item), and recreational activities (1 item), for a total of 10 items, each having 5 options for the evaluation of demand (really not required, not required, neutral, required, very much required) and status (very dissatisfied, dissatisfied, neutral, satisfied, very satisfied).

#### Questionnaire for medical staff

The questionnaire included four parts: personal basic information, understanding of the geriatric healthcare industry, training experience, and required training. Personal basic information included age, gender, level of education, work title, number of years worked, etc. Questions about their understanding of the geriatric healthcare industry included 8 items: degree major subject, whether courses associated with care of the elderly were studied, satisfaction with their job and income, why this profession was chosen, what training the respondent considered was still required, and the types of professionals and services urgently required. Questions about training experience included 7 items, such as whether the employee had received training relevant to geriatric healthcare, content of the training, whether relevant assessment had been conducted, and the type of assessment performed. Questions about training requirements included 8 items, including whether additional training is expected, what the anticipated training consists of, the manner in which it should be conducted, problems of the current training, and suggestions for future training.

#### Questionnaire for nursing staff

The questionnaire included general information, current working arrangements, training received and training required. General information included age, gender, level of education, and marital status. Work arrangements included whether certification had been obtained, the level of certification, reasons for entering the nursing profession, type of work performed, years worked, daily work schedule, and their evaluation of the difficulty of work. Questions about training experience included 15 items, including whether any job-related training had been received, the method, content, frequency, and purpose of the training, and any problems in the current training. Training needs included 8 items, such as the extent of training required, how the time required for training was expected to be scheduled, the expected length, mode, frequency, content of training, and suggestions for future training.

### Data collection

Investigators used “Questionnaire Star” software to conduct the online surveys after they had received unified training. They explained the purpose and significance of the study to respondents face to face who then provided signed informed consent after agreeing to participate in the study. Respondents then completed the relevant questionnaire. The questionnaires were designed to save a respondent’s answers automatically and to continue from the same point in the questionnaire if the survey was reopened, after scanning the relevant code. Answers to all questions were required prior to submission.

### Statistical analysis

All data were analyzed with SPSS v22.0 software. Non-normally distributed measurement data were expressed as medians, for example, working years, working days per week of nursing stuff, and the numbers of disabled geriatric individuals they cared for each day. Frequencies and percentages were used for the descriptive statistics of numerical data.

## Results

### General information

A total of 589 care recipients were included in the study, including 501 (85.06%) in nursing homes, and 88 (14.94%) in geriatric hospitals, of which 519 (88.12%) were aged between 65 and 90. In total, 532 (90.32%) had at least one chronic disease. The self-care dependency of 199 subjects (33.79%) was mild, moderate in 144 (24.45%), and severe in 123 (20.88%). From the self-rated scoring of health status, 286 cases (48.56%) scored below 60, 209 (35.48%) scored 61-80, and 94 (15.96%) scored 81-100. The three greatest expenses for care receivers were the fixed expenses of the nursing institution, expenses required to employ family nurses, and their everyday living expenses (clothing, meals, etc.).

A total of 415 healthcare staff were included in the study, including 144 doctors (34.7%), 213 nurses (51.33%), 34 rehabilitation instructors (8.19%), and 24 pharmacists and nutritionists (5.78%) The majority (82.65%) were women. A total of 43.86% were 30 to 39 years of age, 54 (13.1%) had a Masters degree or above, 249 (60%) had a Bachelors degree, 81 (19.52%) had a junior college certificate, while 31 (7.47%) had received only technical secondary school education or below. There were 157 members of staff (37.83%) that had worked for 5 years or less, 105 (25.3%) had worked for 5-10 years, while 153 (36.87%) had worked for 10 years or more. In total, 256 (5.38%) had a primary professional title, 125 had (30.12%) an intermediate professional title, and 37 (8.92%) had gained a senior professional title.

A total of 372 nursing staff were included in the study who were mainly female, accounting for 89% of the total. The staff was mostly 40 to 60 of age, 87.4% of the total. A total of 32 (8.6%) had received a university education or above, 215 (57.8%) had only a junior high school education, 83 (22.3%) had a technical secondary school / senior high school education, while 42 (11.3%) had only a primary school education or below. In total, 53.5% had obtained a work qualification certificate, while 74.4% had a junior title. The median length of working was 2 years, most worked 5 days per week, and the median number of disabled geriatric individuals they cared for was 6. Daily work included providing everyday living care services (92.7%), nursing assistance (83.6%), and psychological or spiritual support (69.4%). Their evaluation of the difficulty of work was moderate.

### Requirement for healthcare services

In total, 88.96% of care recipients wanted support to be received in nursing homes, apartments for the older population, medical or nursing service centers, or other institutions. The five most common reasons for choosing that retirement category, ranked in order, were the quality of care services (88.12%), availability of professional medical services (82.00%), quality of facilities (80.65%), cost (34.30%), and proximity to home (22.41%). The extent of training required was calculated by dividing the number of questionnaires scored as “required” and “very much required” by the total number, and the extent of satisfaction by dividing the number of questionnaires scored “satisfied” and “very satisfied” by the total number. The service with the greatest demand and satisfaction were regular visits by medical staff. The results for other services are displayed in Table 1.

**Table 1:** Extent of demand for healthcare services and satisfaction of recipients

	Demand (%)	Satisfaction (%)
Medical staff making regular visits	87.27	87.27
Assistance with referrals and emergency ambulance services	82.85	84.55
Regular physical examination and health education activities	82.51	83.36
Providing professional rehabilitation assistance consultation, guidance, training	82.51	81.83
Volunteer accompaniment, environmental adaptation, emotional counseling	81.66	80.98
Providing and equipping with appropriate rehabilitation equipment	77.25	78.78
Assistance in maintaining personal and household hygiene	76.06	87.10
Escrow goods, agency, and other services	69.78	83.02
Assistance in feeding, medical treatment, and other activities	69.44	85.74
Organizing cultural and entertainment activities	68.08	79.63

### Training status and requirements of medical staff

From the questionnaires of medical staff, the reasons for choosing the geriatric healthcare profession were that the role matched their university medical training (57.83%), optimism about the job prospects of working with older people (41.44%), post-transfer entry (9.16%), and directed training (8.43%). The proportion satisfied with their job was 74.2% while 37.1% were satisfied with their income. In total, 59.04% studied geriatric medicine/nursing in their education, 81.45% received training after starting their job, while the principal method of receiving the relevant training was by lectures in units (66.51%), with 75.42% requiring additional training in geriatric healthcare. Suggestions included paying attention to the psychological health of care receivers, and governmental and relevant institutions providing greater support and being able to learn advanced technologies both nationally and internationally, etc. The expected content and methods of training are detailed in Table 2.

**Table 2:** Training needs of medical staff

	Number	Proportion (%)
1. Your requirements for training related to geriatric healthcare:		
Really not required	4	0.96
Not required	5	1.2
Neutral	93	22.41
Required	232	55.9
Very much required	81	19.52
2. Do you think the training satisfied your needs?		
Very dissatisfied	6	1.45
Dissatisfied	20	4.82
Neutral	109	26.27
Satisfied	194	46.75
Very satisfied	86	20.72
3. Can the existing training solve problems encountered during work?		
Completely cannot solve	1	0.24
Mostly cannot solve	17	4.1
Neutral	121	29.16
Mostly can solve	215	51.81
Completely can solve	61	14.7
4. Should training content be increased?		
Yes	302	72.77
No	113	27.23
5. What training content should be added?		
Comprehensive geriatric assessment	198	47.71
Geriatric rehabilitation healthcare	186	44.82
Chronic disease management	185	44.58
Geriatric nutrition	172	41.45
Geriatric nursing technology	148	35.66
Geriatric physiology	147	35.42
Humanistic geriatric care	143	34.46
Health education	137	33.01
Risk management and prevention	115	27.71
Professional ethics and laws and regulations	84	20.24
Operation management	63	15.18
Scientific research	60	14.46
6. How should training be conducted?		
Self-study online video courses	297	71.57
Work hours used to focus on training	194	46.75
Intensive training during spare time	112	26.75
7. What are the current training problems?		
Lack of training opportunities	189	45.54
Single training methods	159	38.31
Duration of training time or location is inappropriate	158	38.07
Training content is not practical	78	18.8
Trainers are not sufficiently professional	46	11.08
Other	22	5.3

### Training status and the requirements of nursing staff

During employment, 83.6% of nursing staff participated in relevant training, with training provided usually once a month, for 62.4% of staff. The principal purpose of the training was to improve work skills (87.1%) and to obtain relevant practicing certification (53.4%). The training mainly included everyday living care services (92.0%), nursing assistance (83.0%), professional ethics (72.7%), communication skills (72.7%), rehabilitation services (66.6%), psychological support (61.7%), and laws and regulations (62.4%). In total, 85.6% thought the training was easy to understand, 53.4% thought the training was helpful for work, although only 38.9% were very satisfied with the training. Problems with the current training were principally the lack of training opportunities (40.8%) and inappropriate duration or location of training (35.7%). The main reasons for not participating in training were the inappropriate time the training was provided (46.2%), and the need to pay for training expenses (38.5%). Additional training was required by 86% of respondents. The required training and the methods of delivery are detailed in Table 3.

**Table 3:** Training requirements for nursing staff

	Number	Proportion
1. Requirments for training:		
Very much not required	1	0.3
Not required	7	1.9
general	44	11.8
Required	225	60.5
Very much required	95	25.5
2. How should training be provided?		
One-time training	81	21.8
Phased training	291	78.2
3. During work, the frequency of ongoing training that should be provided is:		
Once a month	127	34.1
Once a quarter	143	38.4
Once every six months	79	21.2
Once a year	23	6.2
4. After work, the duration of training should be:		
Less than 30 min	56	15.1
30-60 min	210	56.5
61-90 min	88	23.7
61-90 min	13	3.5
More than 120 min	5	1.3
5. What type of training is most required?		
Everyday living assistance (catering, cleaning, bathing, travel, etc.)	290	78
Nursing assistance (basic nursing, such as turning over, skin protection, etc.)	263	70.7
Rehabilitation services (assisting in rehabilitation training)	25	67.2
Professional ethics (workflow, professional standards, professional norms)	273	73.4
Laws and regulations (relevant laws and regulations for nursing staff)	258	69.4
Communication skills	265	71.2
Mental and psychological support	213	57.3
6. How should training be conducted?		
Practical training	312	83.9
Theoretical lecture training	285	76.6
Network teaching and training	258	69.4
Colleague experience sharing	180	48.4
On site observation	120	32.3

## Discussion

The present study demonstrates that the older population has a greater demand than currently provided for medical and healthcare services, such as regular visits, assistance for referrals, emergency assistance services, and regular physical examination. The availability of professional medical services ranked second as a reason for choosing the category of institution selected for the provision of care services, possibly related to the majority of residents having chronic diseases and poor physical health. In this survey, 90.32% of care recipients had chronic diseases, 79.12% had an inability to administer self-care, while almost half had a self-rated health status score lower than 60 points. These results are consistent with the study of Han Hu [18] and demonstrates that China has the largest number of their aging population in need of long-term care, with 70% having a chronic disease. Secondly, services in high demand are professional rehabilitation assistance consultation, guidance, and training, etc. As a group with a high incidence of physical dysfunction and disablement, old people often require professional rehabilitation training to promote physical recovery, reflected in the survey results, which demonstrated that there was a high demand for rehabilitation guidance services [19, 20]. The requirement for spiritual and psychological consolation was as high as 81.66%, indicating that the older population has a greater requirement for care services. In addition to traditional financial support, assistance with daily living and other old-age care activities, spiritual problems of the older population have become increasingly prominent. Aging individuals not only require physical health, but also have diverse spiritual needs, spiritual and interpersonal communication requirements, cultural entertainment, etc., that reflect their own values [21, 22]. In terms of mental care, geriatric care institutions provide environmental adaptation, care visits, life companionship, emotional and psychological counseling, and other services to improve the overall quality of life of older people [23].

At present, there are more than 40 million disabled or semi-disabled geriatric individuals in China, and so there is increasing demand for caregivers [24]. There is an urgent need for healthcare professionals with knowledge of medicine, psychology, physiology, sociology, etc. [25]. In the present survey, it was found that 78.3% of medical staff in healthcare institutions have training needs, and 72.77% require increased training content. Previous studies have also shown that the current training cannot meet the growing multi-level and diversified needs of geriatric health and care services [26, 27]. It is also challenging to provide high-quality nursing, medical rehabilitation, spiritual comfort, and other services, so it is necessary to increase the training for comprehensive assessment, rehabilitation, and healthcare, chronic management, geriatric nutrition, humanistic care, etc. In total, 83.6% of geriatric nursing staff have participated in on-the-job training, but only 53.4% thought that the content was helpful for work, and only 38.9% were very satisfied with the training. In addition to the training content, these observations may also be related to the methods and arrangements for training. Both professional medical staff and nursing staff suggest that more practical training should be provided. Such training can improve the effectiveness of learning, strengthen memory, and when combined with normal work routines, improve the quality of clinical nursing [28, 29]. In terms of training arrangements, the most requested frequency of training for caregivers was once per quarter, using phased training, the duration of which should be between 30 and 60 min. It is clear that formation of geriatric healthcare talent teams is a long process, which should be gradual, not too rapid, and utilizing processes that do not ignore quality.

The level of education of nursing staff in the present survey was generally low, with only 8.6% having a college degree or above, and only 53.5% having work qualification certificates. This may be related to a reduction in the employment threshold caused by the considerable lack of geriatric nursing staff. According to surveys, more than 10 million care staff are required in China, while the actual number of employees is less than 1 million [30]. In this regard, the government can, through the use of policy guidance, attract more high-quality talent to enter the field of geriatric healthcare, improve the overall professional level of the industry, and establish and improve vocational qualification certification and training systems. Professional and technical personnel engaged in healthcare should have obtained the relevant healthcare qualifications, while those without those qualifications should instead have the corresponding job training certification. In addition, the financial and social status of the employees should be improved so that occupational attraction is improved. The salaries and benefits of healthcare workers should be linked to their professional skill levels and the duration and difficulty of the work, so that those who are highly skilled earn more, as should those that work hard. On the other hand, colleges and universities are strongly advocated to offer major degree subjects related to geriatric healthcare, introduce incentive schemes for healthcare personnel training, attract students to study geriatric healthcare through a reduction in tuition fees, and provide scholarships and employment subsidies, etc., to improve the reserve of talent.

To make the results more representative, we selected research objects from different settings. However, the number of participants was unbalanced. There were more participants in nursing homes than in communities or geriatric hospitals, which may have led to selection bias. In addition, Although the study included as many participants as possible, all were from northeast China, and so the findings may not be generalizable to other population centers. There is still a need for additional cross-regional, large-sample, multi-center studies to better understand the demand for care services for the older population and the training needs of their care workers.

## Conclusions

As the demands of the aging population are clearly increasing, the demand structure has been transformed from survival to one of development. The diverse needs of old people have a greater requirement for cultural knowledge, with greater business skills, service level, qualifications, and professional quality of healthcare workers, so training healthcare service talent is urgently required to build healthcare service capacity. The Government should consider enhanced top-level design, and provide guidance so that healthcare service personnel training is developed. To improve the effectiveness and quality of the training, various methods can be explored, such as scenario simulation and practical demonstrations, etc., closely combined with clinical work, with multi-disciplinary training that is based on the needs of older people. In addition, cooperation with colleges and universities should be encouraged to strengthen the reserve of talent and cultivate the professional quality of geriatric healthcare personnel.

## Data Availability

The datasets used and analyzed during the current study are available from the corresponding author upon reasonable request.
